# Brain–blood amino acid correlates following protein restriction in murine maple syrup urine disease

**DOI:** 10.1186/1750-1172-9-73

**Published:** 2014-05-08

**Authors:** Kara R Vogel, Erland Arning, Brandi L Wasek, Sterling McPherson, Teodoro Bottiglieri, K Michael Gibson

**Affiliations:** 1 Experimental and Systems Pharmacology, College of Pharmacy, Washington State University, 412 E. Spokane Falls Blvd., Pharmaceutical and Biomedical Sciences Building, Room 347, P.O. Box 1495, 99210-1495 Spokane, WA, USA; 2 Institute of Metabolic Disease and Baylor Research Institute, Baylor University Medical Center, Dallas, TX, USA; 3 College of Nursing, Washington State University, Spokane, WA, USA

**Keywords:** Maple syrup urine disease (MSUD), Branched-chain keto-acid dehydrogenase (BCKDH) complex, Mouse model, Large neutral amino acid transporter (LAT-1), Branched-chain amino acids (BCAAs), Phenylketonuria (PKU), Large neutral aminoacidopathies, Dietary protein restriction

## Abstract

**Background:**

Conventional therapy for patients with maple syrup urine disease (MSUD) entails restriction of protein intake to maintain acceptable levels of the branched chain amino acid, leucine (LEU), monitored in blood. However, no data exists on the correlation between brain and blood LEU with protein restriction, and whether correction in blood is reflected in brain.

**Methods:**

To address this question, we fed intermediate MSUD mice diets of 19% (standard) and 6% protein, with collection of sera (SE), striata (STR), cerebellum (CE) and cortex (CTX) for quantitative amino acid analyses.

**Results:**

LEU and valine (VAL) levels in all brain regions improved on average 28% when shifting from 19% to 6% protein, whereas the same improvements in SE were on average 60%. Isoleucine (ILE) in brain regions did not improve, while the SE level improved 24% with low-protein consumption. Blood-branched chain amino acids (LEU, ILE, and VAL in sera (SE)) were 362-434 μM, consistent with human values considered within control. Nonetheless, numerous amino acids in brain regions remained abnormal despite protein restriction, including glutamine (GLN), aspartate (ASP), glutamate (GLU), gamma-aminobutyric acid (GABA), asparagine (ASN), citrulline (CIT) and serine (SER). To assess the specificity of these anomalies, we piloted preliminary studies in hyperphenylalaninemic mice, modeling another large neutral aminoacidopathy. Employing an identical dietary regimen, we found remarkably consistent abnormalities in GLN, ASP, and GLU.

**Conclusions:**

Our results suggest that blood amino acid analysis may be a poor surrogate for assessing the outcomes of protein restriction in the large neutral amino acidopathies, and further indicate that chronic neurotransmitter disruptions (GLU, GABA, ASP) may contribute to long-term neurocognitive dysfunction in these disorders.

## Background

Maple syrup urine disease (MSUD; branched chain ketoacid dehydrogenase (BCKDH) deficiency) and phenylketonuria (PKU) comprise the large neutral aminoacidopathies. Untreated MSUD results in accumulation of the branched-chain amino acids (BCAAs) leucine (LEU), valine (VAL) and isoleucine (ILE) [1-4]. Additionally, induction of catabolism will induce release of BCAAs from muscle and increase the BCAA’s corresponding α-keto acids (BCKAs), including α-ketoisocaproate (KIC, from LEU), α-keto-β-methylvalerate (KMV, from ILE) and α-ketoisovalerate (KIV, from VAL), all of which can freely traverse the blood–brain barrier (BBB) and transaminate to yield the corresponding BCAAs [5]. Treatment of MSUD requires strict protein restriction combined with BCAA supplementation to levels compatible with adequate development and growth. Although dietary intervention prevents the severe developmental delays associated with chronic hyperleucinemia, it nonetheless presents challenges that include maintenance and adherence to diet, and mounting evidence for long-term deficits in neurocognition despite acceptable metabolic control determined in blood [6-16]. Additionally, over restriction of dietary amino acid intake may have untoward consequences. The preceding discussion suggests that while prudent, dietary treatment alone for MSUD may be suboptimal.

The pathophysiology of MSUD has been extensively reviewed [17]. Episodic increases in blood BCAAs occur when muscle degrades protein in response to physiological stress [18-20], also increasing the level of corresponding BCKAs. Increased muscle BCKAs induces reversal of cytosolic transaminases and depletes tissue levels of other amino acid nitrogen donors. As LEU exits muscle and other tissues via the large neutral amino acid transporter (LAT-2) [21], heteroexchange mechanisms stoichiometrically drive import of other amino acids and increase the relative LEU blood concentration. At the BBB, elevated BCAAs may saturate the LAT-1 (see below) transporter and block uptake of other large neutral amino acids (LNAA; including phenylalanine (PHE), tyrosine (TYR), methionine (MET); tryptophan (TRP); and histidine (HIS)). Increased blood BCKAs enter the brain via the monocarboxylate transporter (MCT) and reverse flux through cerebral transaminases, depleting brain glutamate (GLU), glutamine (GLN) and gamma-aminobutyric acid (GABA) (among others), while enhancing production of LEU and α-ketoglutarate [5,22-25]. These disruptions in brain amino acid homeostasis may also be accompanied by disruptions of oxidative phosphorylation, elevated cerebral lactate level and oxidative damage [20,26].

As noted above, LNAAs cross the BBB on the LAT-1 transporter, and accumulation of offending amino acids such as the BCAAs in MSUD can result in exclusion of other LNAAs from the brain, although this has not been rigorously evaluated in either patients or an animal model of MSUD. Despite the specificity of the LAT-1 for LNAAs, most brain amino acid transport systems display broad overlap in the amino acids transported [3,4,27,28]. Here, we have employed a murine model of MSUD to address the question of whether control of BCAA level, as monitored in blood, is reflected by similar correction in the brain. Several novel therapeutic approaches to PKU have emerged recently [29], yet with the exceptions of phenylbutyrate administration (which selectively lowers circulating BCAAs) [30] or orthotopic liver transplantation for the most severe forms of MSUD [8,15,31,32], few therapeutic advances have emerged in MSUD. The current report addresses our hypothesis, and for the first time verifies in a murine model of MSUD that assessment of metabolic control using blood studies is a poor surrogate for disturbances of amino acid homeostasis in the brain.

## Methods

### Animal subjects

The intermediate MSUD murine model (*imsud* mice) has been described, and manifests ~ 6% of residual BCKDH enzyme function [1,2,33,34]. Animal subjects were genotyped by PCR amplification. Subjects of both genders were employed, ages 10–20 days of life. Subjects were allowed ad libitum access to chow and water throughout experimentation. All animal experimentation was performed with IACUC approval (ASAF 4232–007).

### Diets employed

We performed pilot studies with the Teklad TD.90016 diet (6.9% casein = 6% protein; low protein) since it has been employed previously for maternal protein restriction in rodents [35]. In the latter studies, the induction of serine-metabolizing enzymes was characterized in mice receiving 2, 6 and 18% protein. Based upon empiric considerations, we felt that 2% protein would be too stringent in *imsud* mice and lead to catabolic crisis. Teklad custom diets are isocaloric and balanced for nitrogen level. The corresponding Teklad TD.91353 diet (19% protein) was used as control diet. Animals were on diet for 8–18 days prior to sacrifice. Power analyses indicated that n = 6 subjects of each genotype (*imsud*^*+/+*^ and *imsud*^*-/-*^*)* would be sufficient to obtain statistically significant outcomes. For brain region analyses (see below), we frequently had n = 5-6 subjects per diet and genotype, but the smaller size of mutant subjects often led to insufficient blood for analytical studies, thereby leading to smaller n values for sera (SE) and enhanced data variability. At study conclusion, subjects per genotype were n = 3-7 for SE and n = 4-8 for brain.

### Tissue harvesting and analytical methodology

At appropriate time points, animal subjects were euthanized, and blood collected by cardiac puncture. The brain cavity was rapidly accessed on an ice-cold glass plate, with parietal cortex (CTX), striata (STR), and cerebellum (CB) rapidly excised. Harvested tissues and sera following centrifugation were stored at – 80°C until analyses. LNAAs and other amino acids were determined with an ultraperformance liquid chromatography (UPLC) MassTrak amino acid system as previously described [36]. All values in SE are presented in units of μmol/L, wherease all values in brain regions are presented in units of nmol/gr tissue.

### Statistical analyses

In prior studies, we had observed depletion of GLU, GLN, ASP, serine (SER), alanine (ALA) and GABA, with elevated glycine (GLY) in brain of *imsud* mice. However, those studies were performed in whole brain [1,2,33,34], potentially leading to artifact from mixing of regions with high vs. low metabolite levels. In the current study we assessed discrete brain regions. Accordingly, we had no insight whether to expect brain regional amino acids to be constant or different. Thus, we initially employed a two-way ANOVA to assess correlations between brain region and protein intake, and interaction of these parameters, accompanied by a Holm-Sidak’s multiple comparisons test. Our primary statistical analyses employed a two-tailed *t* test within genotype as a function of protein consumption. Both statistical evaluations are presented in all figure legends. Comparisons of amino acid concentrations between blood and brain assumed a density of brain tissue of ~1 g/ml [37].

## Results

**Figure 1:**
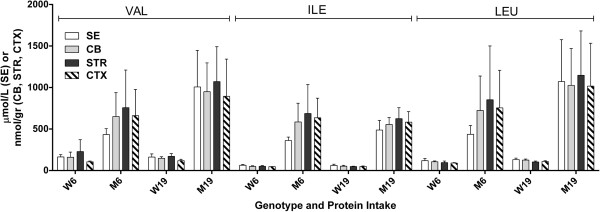
**Branched chain amino acids in sera (SE), cerebellum (CB), striata (STR) and cortex (CTX) as a function of protein intake (6 or 19%) and genotype (w, wild-type; m, mutant).** Two-way ANOVA (parameters: protein intake, tissue), p = ns for tissue and interaction, p < 0.05 for protein intake. Two-tailed *t*-test, p < 0.05 for all mutant vs. wild-type (not shown).

For *imsud* mice, all BCAAs were significantly increased in comparison to littermate controls, verifying the utility of the model (Figure 1). With decreased protein consumption, LEU in mutant SE decreased from 1072 (504) (standard deviation shown in parenthesis after mean) to 436 (107) μmol/L, while the same decreases in mutant were 1026 (444) to 723 (415) in CB, 1147 (535) to 853 (648) in STR and 1017 (516) to 755 (452) in CTX (brain regions: nmol/gr tissue). ILE changes in mutant mice with decreased protein were 488 (115) to 362 (41) μmol/L in SE, 555 (84) to 585 (226) in CB, 625 (132) to 687 (349) in SE and 583 (126) to 636 (235) in cortex (nmol/gr tissue). For VAL, comparable changes were 1007 (441) to 434 (71) μmol/L in SE, 950 (347) to 651 (288) in CB, 1071 (421) to 758 (453) in STR and 894 (448) to 661 (351) in CTX (nmol/gr tissue).

**Figure 2:**
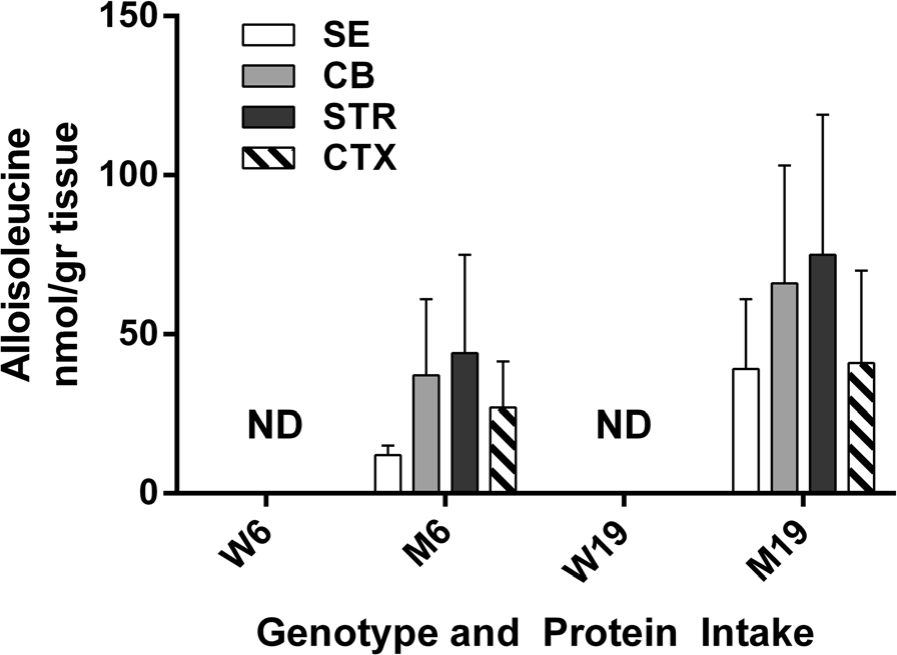
**Alloisoleucine levels (see Figure**1**for details). **Two-way ANOVA, p < 0.05 for protein intake, p = ns for tissue and interaction. Two-tailed *t*-test, p = ns between mutant and wild-type as a function of protein intake.

Within tissues (SE, CB, STR and CTX), there was no significant improvement in BCAAs with lowered protein intake, although the trend in SE BCAAs approached significance (*t-*test, 2-tailed, p = 0.074-0.125 vs. CB, STR and CTX, p = 0.210-0.789), but variation in SDs (occasional low SE quantity for selected animals) was problematic. Alloisoleucine (ALLO, a pathognomonic biomarker for MSUD) showed similar trends (Figure 2). The changes for ALLO in mutants with decreased protein consumption were 39 (22) to 12 (3) μmol/L in SE, 66 (37) to 37 (24) in CB, 75 (44) to 44 (31) in STR, and 41 (29) to 27 (15) in CTX (brain values, nmol/gr protein). As expected, ALLO was undetectable in wild-type mice with either diet. There was no significant difference in any tissue with decreased protein intake for ALLO in mutant mice.

**Figure 3:**
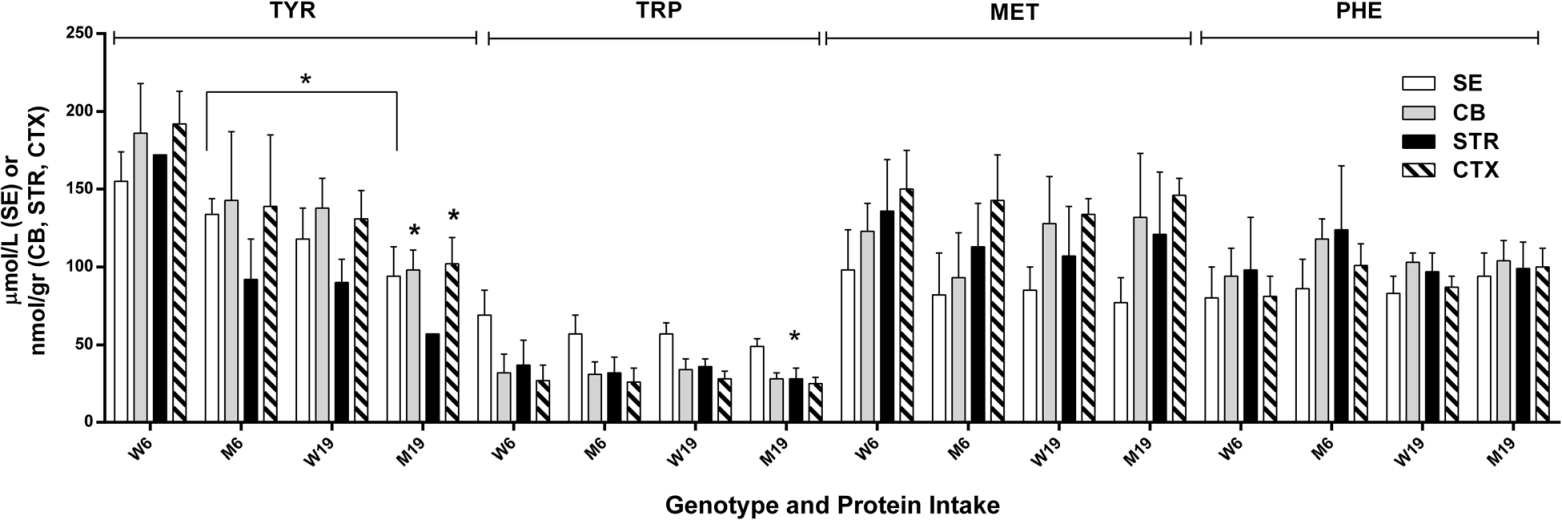
**Other large neutral amino acids (tyrosine, tryptophan, methionine, phenylalanine) as a function of protein intake and genotype (see Figure**1**for details).** Two-tailed ANOVA, p < 0.05 for both protein intake, tissue and interaction. Two-tailed *t*-test (*p < 0.05), comparing M19 and W19 in CTX and CB (TYR) and STR (TRP). Also, p < 0.05 for TYR in SE (M6 vs M19).

We then turned our attention to the other LNAAs (MET, PHE, TYR and TRP) in the same regions (Figure 3). For TYR, changes for mutant mice with decreased protein intake were 94 (19) to 134 (10) μmol/L in SE, 98 (13) to 143 (44) in CB, 57 (n = 2)-92 (26) in striata and 102 (17) to 139 (46) in CTX (brain, nmol/gr tissue). For TRP, the same values for mutant were 49 (5) to 57 (12) μmol/L in SE, 28 (4) to 31 (8) in CB, 28 (7) to 32 (10) in STR and 25 (4) to 26 (9) in CTX. For MET, these values were 77 (16) to 82 (27) μmol/L in SE, 132 (41) to 93 (29) in CB, 121 (40) to 113 (28) in STR and 146 (11) to 143 (29) in CTX. For PHE, the corresponding values were 94 (15) to 86 (19) μmol/L in SE, 104 (13) to 118 (13) in CB, 99 (17) to 124 (41) in STR and 100 (12) to 101 (14) in CTX (nmol/gr tissue).

Elevated BCAA levels had no substantial effect on TRP, MET and PHE levels in any brain region, although SE TYR was improved (increased in mutant mice; Figure 3) with decreased protein consumption (without a corresponding correction in brain regions). This finding lends credence to the hypothesis that correction of blood amino acid content may not reflect corresponding corrections in brain. Conversely, compared to control mice receiving identical diet, levels of TYR or TRP were depleted in CB, STR and CTX with high protein intake, but these depletions were not consistent across different brain regions (Figure 3).

**Figure 4:**
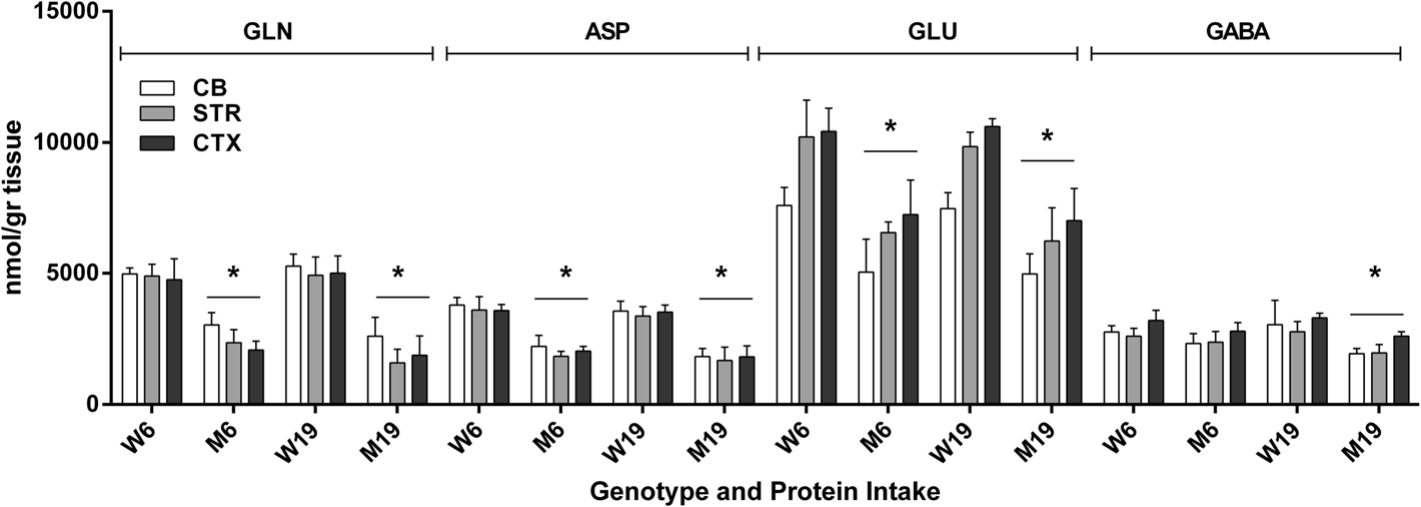
**Neurotransmitter amino acids (glutamine, aspartate, glutamate and GABA) as a function of protein intake and tissue (see Figure**1**Legend).** Two-way ANOVA, p < 0.05 for tissue, protein intake and interaction. Two-tailed *t*-test, *p < 0.05 for all mutant vs. wild-type under both protein intake regimens.

**Figure 5:**
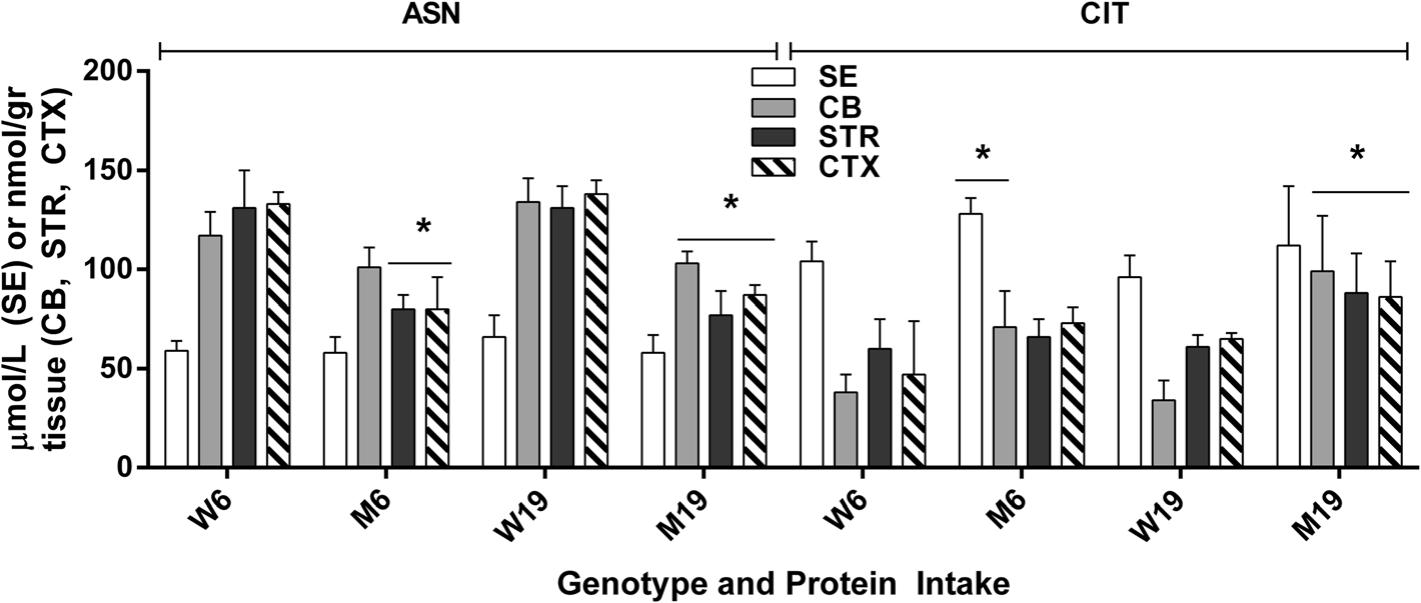
**Asparagine and citrulline level as a function of protein intake and tissue.** Two-way ANOVA, p < 0.05 for protein intake and interaction, p = ns for tissue. Two-tailed *t*-test, *p < 0.05 between genotype with identical protein intake.

**Figure 6:**
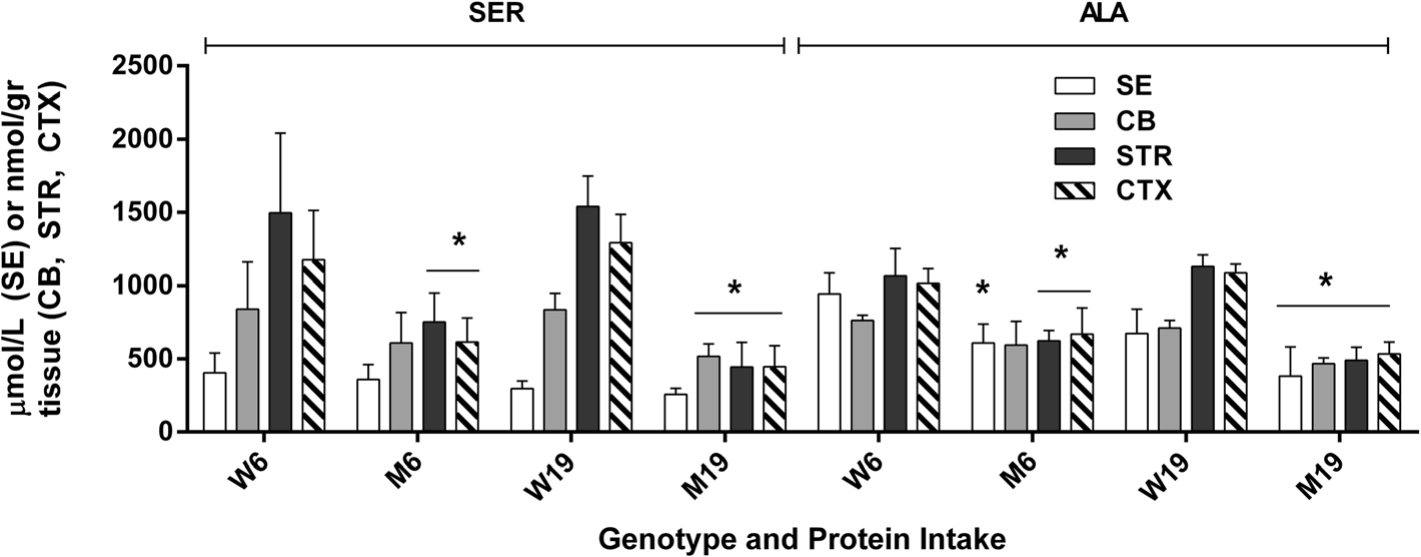
**Serine and alanine levels as a function of protein intake and tissue.** Two-way ANOVA, p < 0.05 for protein intake, tissue and interaction. Two-tailed *t*-test, *p < 0.05 for genotype with identical protein intake.

We next surveyed all remaining physiological amino acids across brain regions, and observed significant abnormalities in GLN, ASP, GLU, GABA, ASN (asparagine); CIT (citrulline), SER and ALA (Figures 4, 5 and 6). For comparison purposes, concentrations for GLN, ASP and GLU are not depicted for SE because of the much higher brain levels, which would graphically dwarf SE values, and GABA is not quantifiable in SE. Changes in GLN in mutant brain regions with decreased protein consumption were 2590 (726) to 3021 (477) in CB, 1575 (516) to 2342 (503) in STR and 1862 (739) to 2064 (336) in CTXD (nmol/gr tissue). For ASP, the same changes with decreased protein were 1818 (303) to 2206 (420) in CB, 1662 (517) to 1829 (183) in STR and 1808 (420) to 2021 (187) in CTX; for GLU, 4973 (774) to 5045 (1263) in CB, 6238 (1264) to 6554 (407) in STR and 7019 (1226) to 7246 (134) in CTX; and for GABA, 1920 (207) to 2317 (378) in CB, 1958 (316) to 2364 (409) in STR and 2595 (171) to 2789 (329) in CTX (nmol/gr tissue).

**Figure 7:**
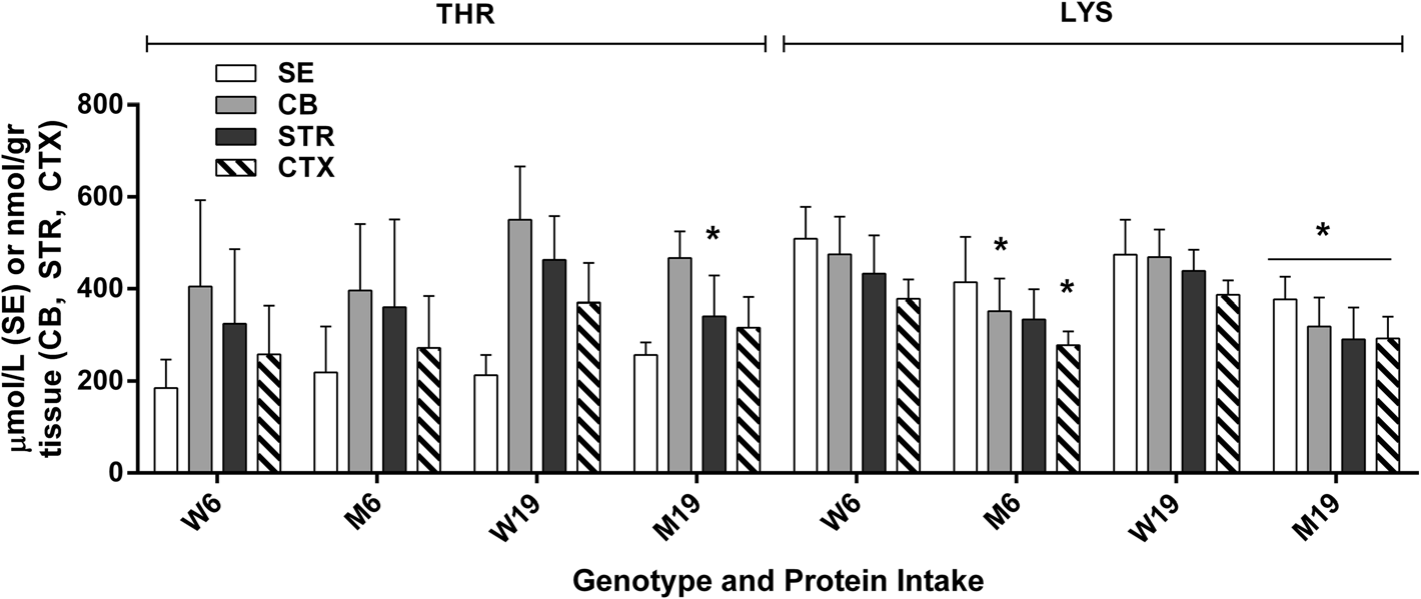
**Threonine and lysine levels as a function of protein intake and tissue.** Two-way ANOVA, p < 0.05 for protein intake, tissue and interaction. Two-tailed *t*-test, *p < 0.05 for genotype with identical protein intake.

With the exception of GABA (which improved with lower protein intake), GLN, ASP and GLU were not improved with lowered protein consumption (Figure 4). Conversely, lowered protein intake normalized the level of ASN in CB and the concentration of CIT in STR and CTX, while both amino acids were significantly increased vs. control during high-protein intake (Figure 5). Similar findings were observed for SER and ALA, in which correction with lowered protein consumption was observed in CB, but not in STR or CTX (Figure 6). Threonine (THR) is often considered a member of the LNAA subgroup, but we found no consistent changes in brain regions, with the exception of a single instance in striata (Figure 7). Conversely, LYS is not considered a LNAA, yet its values were significantly impacted during high-protein consumption (Figure 7).

Since concentrations of several amino acids are much lower in SE vs. brain regions (e.g., Figures 1, 2, and 3), we did not present them graphically, as noted above. We observed no significant changes for GLU and GLN in SE (p > 0.05), yet found brain disruptions that were uncorrected with lowered protein intake (Figure 4). Conversely, in SE we observed a significant difference for ASP (19% diet: *imsud*^*-/-*^, 59 ± 17 (n = 5) vs. *imsud*^*+/+*^*,* 104 ± 21 (n = 7) (p < 0.05), which corrected with 6% diet (*imsud*^*-/-*^, 78 ± 37 (n = 3) vs. *imsud*^*+/+*^*,* 136 ± 90 (n = 5) (p = ns). ASN in SE was within normal limits for *imsud* subjects, despite significant alterations in brain. For CIT, elevations in SE approximated those seen in brain regions (data not shown). SER levels in SE were not abnormal in mutant mice (although they were disrupted in brain regions; Figure 6). Conversely, disruptions of ALA levels in SE mirrored the abnormalities observed in brain (data not shown). Of interest, abnormalities of LYS in SE mirrored the disruptions observed in brain (Figure 7), especially in the instance of high-protein intake, perhaps suggesting that some portion of LYS is trafficked on the large-neutral amino acid transporter.

**Figure 8:**
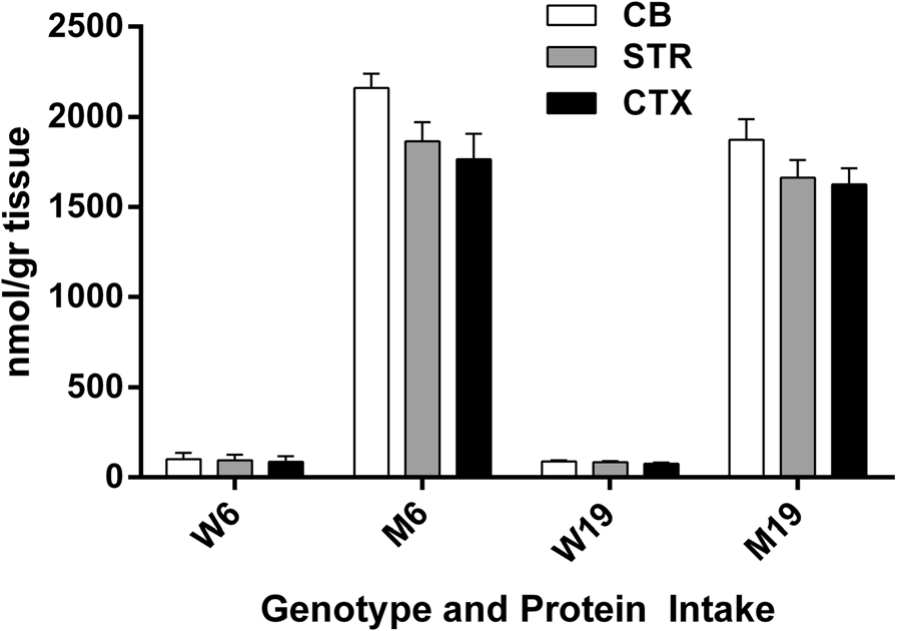
**Phenylalanine levels as a function of protein intake and tissue in phenylketonuric mice.** Two-way ANOVA, p < 0.05 for protein intake, tissue and interaction. All values in mutant mice were significantly increased in comparison to control mice (p < 0.05, two tailed *t*-test; not shown on graph).

**Figure 9:**
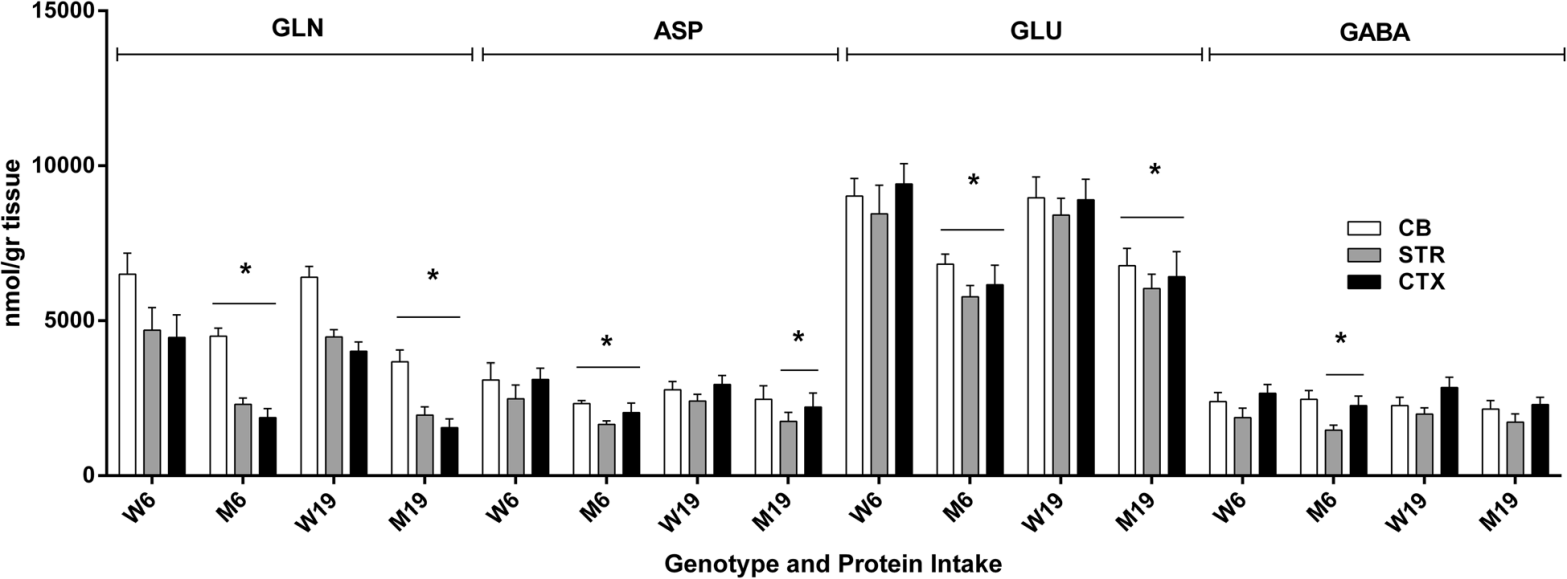
**Glutamine, aspartate, glutamate and GABA as a function of protein intake and tissue in phenylketonuric (PKU) mice.** Two-way ANOVA, p < 0.05 for tissue, protein intake and interaction. Two-tailed *t*-test, *p < 0.05 for mutant vs. wild-type. Note: For GABA in CTX, GABA was also significantly decreased in mutant vs. wild-type mice. In only a single instance did lower protein result in significant correction between mutant populations (GLN in CB).

Depletion of brain GLN, GLU and GABA have been suggested to associate with transamination via accumulated KIC in brain [5]. Accordingly, we predicted that *Pah*^*enu2*^ mice (representing the large neutral aminoacidopathy PKU [3,4]) would have normal GLN, GLU and GABA since KIC is not elevated in this disorder. To address this hypothesis, we developed cohorts of *Pah*^*enu2-/-*^ mice and parallel controls and fed them diets identical to those given to *imsud* mice. We first verified the utility of our PKU model through quantitation of brain PHE levels, which was significantly increased (and not corrected even with 6% protein intake; Figure 8). Unexpectedly, alterations in brain regions for ASP, GLN and GLU in *Pah*^*enu2-/-*^ mirrored those observed in *imsud* mice (Figure 9). These changes in *Pah*^*enu2-/-*^ mice with decreased protein intake were: GLN: 3675 (381) to 4500 (258) in CB, 1964 (261) to 2308 (208) in STR and 1557 (280) to 1876 (297) in CTX (nmol/gr tissue); ASP: 2468 (443) to 2335 (98) in CB, 1758 (293) to 1661 (109) in STR and 2216 (456) to 2044 (306) in CTX; CLU: 6781 (559) to 6833 (320) in CB, 6041 (461) to 5777 (363) in STR and 6427 (810) to 6164 (637) in CTX; GABA: 2152 (279) to 2470 (283) in CB, 1735 (264) to 1470 (168) in STR and 2304 (233) to 2271 (308) in CTX (all nmol/gr tissue). The striking similarity in abnormalities between *imsud*^*-/-*^ and *Pah*^*enu2-/-*^ mice in different regions of the brain, as a function of protein intake (Figure 9), suggests that other factors, in addition to brain keto-acid accumulation, may be at play in the brains of animals with large neutral aminoacidopathies, and most likely in patients as well.

## Discussion

The range of means for BCAAs in SE of *imsud* mice receiving 6% dietary protein was 362-434 μM (Figure 1), consistent with the range of LEU for 13 intermediate MSUD patients under dietary intervention (379 ± 147 μM; [38]) and 282 ± 277 μM for classical MSUD patients [5] considered to be under “metabolic control”. Based on these human data, 6% protein consumption normalized blood BCAAs, yet we observed significant disruption of several other amino acids in the brain despite apparent metabolic control manifested in SE. We hypothesized that reduced levels of GLN, GLU, GABA and ALA in brain regions might be partially explained by KIC accumulation ([5]; see Figures 4 and 6; not measured in the current study), yet similar anomalies in PKU mice argues against this hypothesis (Figure 9). CIT accumulation in *imsud* brain regions likely reflects depletion of ASP, the latter necessary for condensation with CIT to form argininosuccinate within the urea cycle.

The original studies of Pardridge and Oldendorf [39] in the rat indicated that PHE, TRP, MET, TYR, LEU, ILE, VAL and HIS were transported into the brain on the LAT-1, with K_m_ values ranging from ~ 25 to 200 μM (highest affinity, PHE; lowest affinity HIS). Limited data suggests that THR and LYS may also traffic on LAT-1 [3,4,40,41], yet we found no evidence for disrupted THR levels. Conversely, we did detect decreased LYS in brain regions of *imsud* subjects. LYS would be expected to be transported predominantly via the B transporter [3], although there is considerable redundancy in many of the blood–brain transporters.

Long-term neurocognitive deficits are observed in MSUD, even with acceptable metabolic control (as determined in blood), and both animal and human data suggests that these deficits correlate with exclusion of the monoamine precursors TYR and TRP from the brain due to chronic elevations of BCAAs [1,2,25,33,34]. While our studies revealed significant effects on brain TYR with diet in *imsud* subjects, we found no consistent effect on TRP (although we did not quantify monoamine neurotransmitters). Alternatively, our data supports the premise that long-term neurocognitive deficits in MSUD patients may also correlate with depletion of amino acid neurotransmitters (ASP, GLU and GABA). Based upon the preliminary studies in the current report, our next objectives are to investigate LEU-depleted diets in *imsud*^*-/-*^ mice that are appropriately supplemented with ILE and VAL, a diet corresponding to that used in MSUD patients, and to again investigate the amino acid outcomes both regionally in brain and blood. Such studies will yield a better overview of the expected outcomes for a diet more closely resembling that utilized in the clinic. The main conclusions drawn from the current report include:

• The first detailed cross-correlation assessment of amino acids in brain and blood, as a function of total protein intake, and the first analysis of amino acids in discrete regions of the brain of *imsud* mice, including cerebellum, cortex and striata

• A clear demonstration that blood amino acid analysis, and the assignment of metabolic control with respect to BCAA levels, represents only a partial picture of brain amino acid homeostasis

These results further support the concept that dietary intervention in MSUD is likely to be insufficient to avoid neurocognitive deficiencies in patients, underscoring the urgent need for more focused therapeutics in MSUD.

## Abbreviations

SE: Sera; CB: Cerebellum; STR: Striata; CTX: cortex; W6: Wild-type mice receiving 6% protein intake; M6: Mutant mice receiving 6% protein intake; W19: Wild-type mice receiving 19% protein intake; M19: Mutant mice receiving 19% protein intake; imsud-/-: Intermediate maple syrup urine disease mice; Pahenu2-/-: Phenylketonuric mice; LEU: Leucine; ILE: Isoleucine; VAL: Valine; ALLO: Allosisoleucine; SER: Serine; CIT: Citrulline; THR: Threonine; ALA: Alanine; LYS: Lysine; HIS: Histidine; GLN: Glutamine; ASP: Aspartate; GLU: Glutamate; GABA: 4-aminobutyrate; PHE: Phenylalanine; TYR: Tyrosine; MET: Methionine; TRP: Tryptophan; ASN: Asparagine.

## Competing interests

The authors declare that they have no competing interests.

## Authors’ contributions

KRV performed all animal studies, including breeding and dietary intervention, dissection, and assisted with development of the manuscript: EA, BW and TB performed all analytical studies and assisted in drafting the manuscript; SM performed all data analysis, including data evaluation, statistical modeling, and assisted in drafting the manuscript; KMG designed the study, evaluated amino acid data, derived the hypothesis for testing and developed the first draft of the manuscript. All authors read and approved the final manuscript.
